# Supplementary figures and images for: Meiotic recombination modulates the structure and dynamics of the synaptonemal complex during C. elegans meiosis
Source: PLoS Genet. 2017 Mar 24;13(3):e1006670. doi: 10.1371/journal.pgen.1006670 (PMC5384771; doi:10.1371/journal.pgen.1006670)

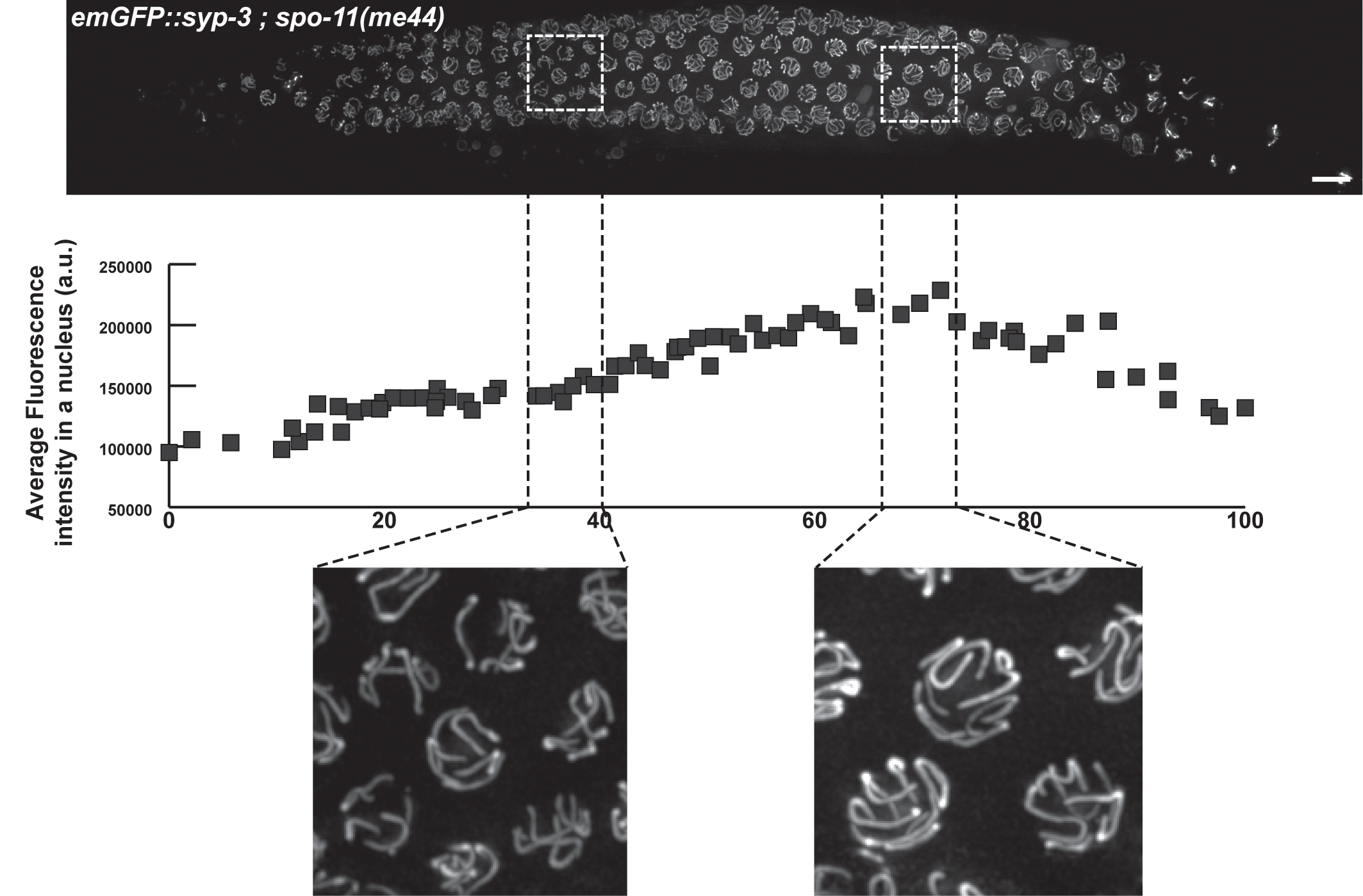

Supplement: S1 Fig — Detection of EmGFP::SYP-3 in the gonad of a live spo-11 mutant worm (top). The graph (middle) represents the evolution of the average fluorescence intensity of a nucleus as a function of its position in the gonad (see Materials and methods). The increase in GFP::SYP-3 signal upon pachytene progression is illustrated in higher magnification fields of early and late pachytene nuclei (bottom). Images are maximum intensity projections of 3D data stacks encompassing whole nuclei. Scale bar represents 10μm. (TIF) [file pgen.1006670.s001.tif]

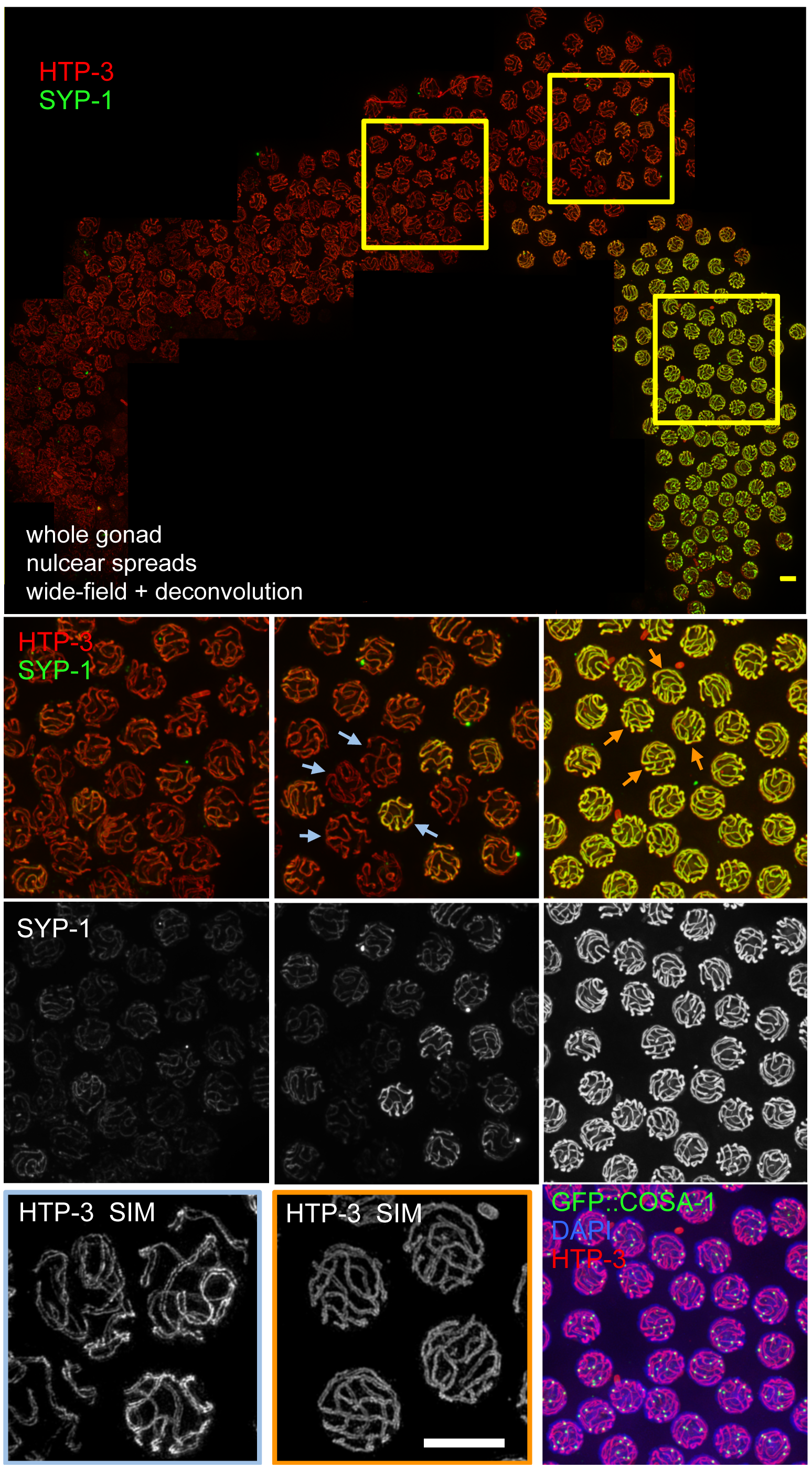

Supplement: S2 Fig — Immunofluorescence images of germ cell nuclei from a C. elegans gonad subjected to detergent-based lysis and partial nuclear spreading using a procedure where the temporal-spatial organization of the gonad can be substantially preserved. Fields of nuclei from early, middle (early-to-late pachytene transition), and late pachytene regions of the gonad, showing that HTP-3 (top row of insets) is retained on chromosome axes following this procedure, whereas SC central region protein SYP-1 (middle row) is largely removed from early pachytene nuclei, yet strongly retained in late pachytene nuclei. The center SYP-1 panels show that adjacent nuclei in the early-to-late pachytene transition region can exhibit different behavior regarding retention/removal of SYP-1. Bottom right inset: late pachytene nuclei with strong SYP-1 retention also exhibit another late pachytene marker, namely the presence of 6 bright GFP::COSA-1 foci marking the sites of CO-designated recombination events. As depicted in the accompanying S3 Fig, the transition to the state where SYP-1 is strongly retained coincides with acquisition of this late pachytene stage marker. Bottom left and center: 3D-SIM images of HTP-3 localization in nuclei from the early-to-late pachytene transition (left, blue outline) and late pachytene (center, orange outline) regions of the gonad; the positions of the depicted nuclei are indicated by blue or orange arrows in the top row of insets. The nuclei where SYP-1 was depleted exhibit wider separation between aligned HTP-3-marked axes than adjacent nuclei where SYP-1 was retained. Images are maximum intensity projections of 3D data stacks; scale bars represent 5 μm. (TIF) [file pgen.1006670.s002.tif]

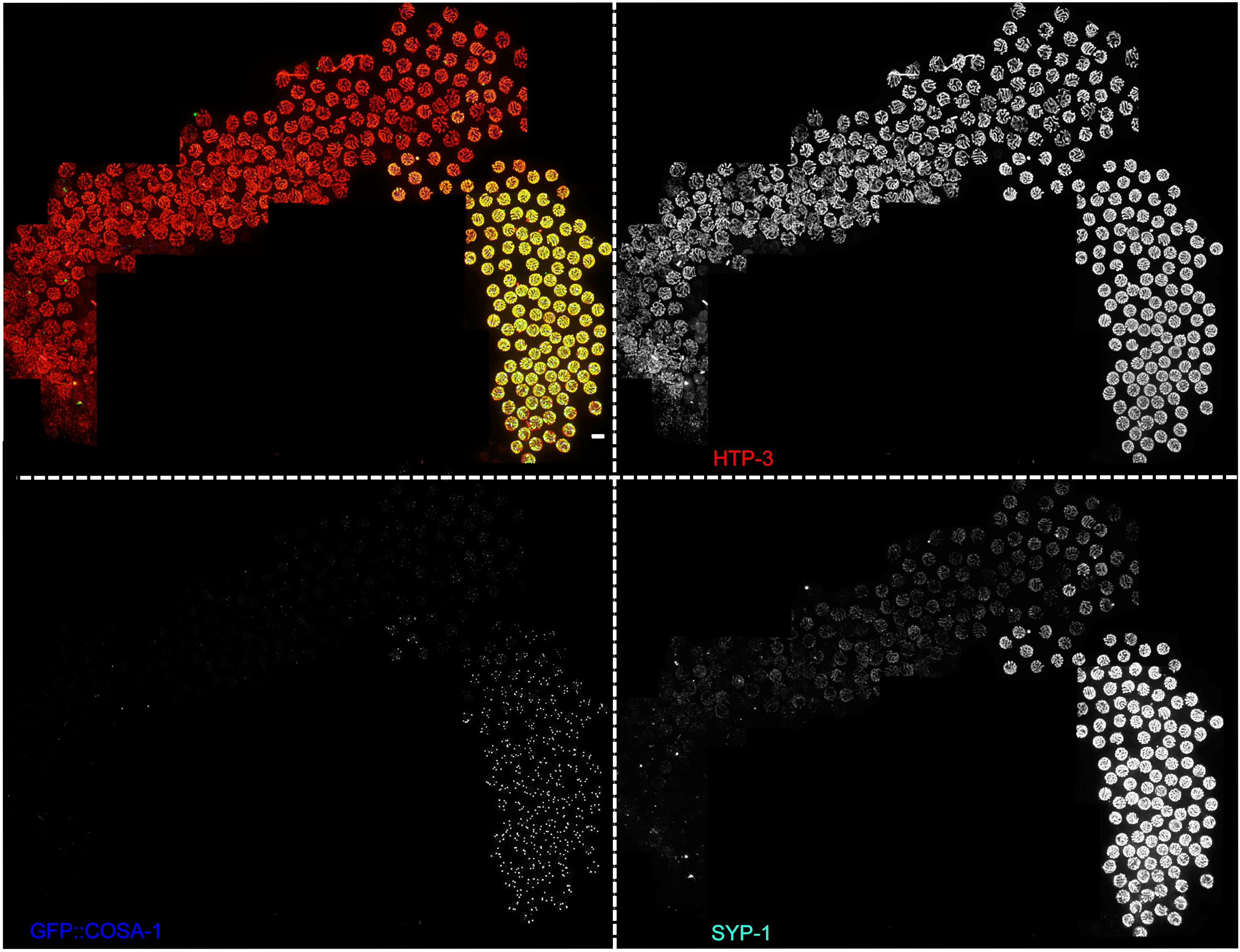

Supplement: S3 Fig — Images of the same spread gonad depicted in S2 Fig, showing HTP-3, SYP-1 and GFP::COSA-1 channels separately to facilitate visualization of the transition from the early to late pachytene state, as indicated by the presence of 6 bright GFP::COSA-1 foci. (TIF) [file pgen.1006670.s003.tif]

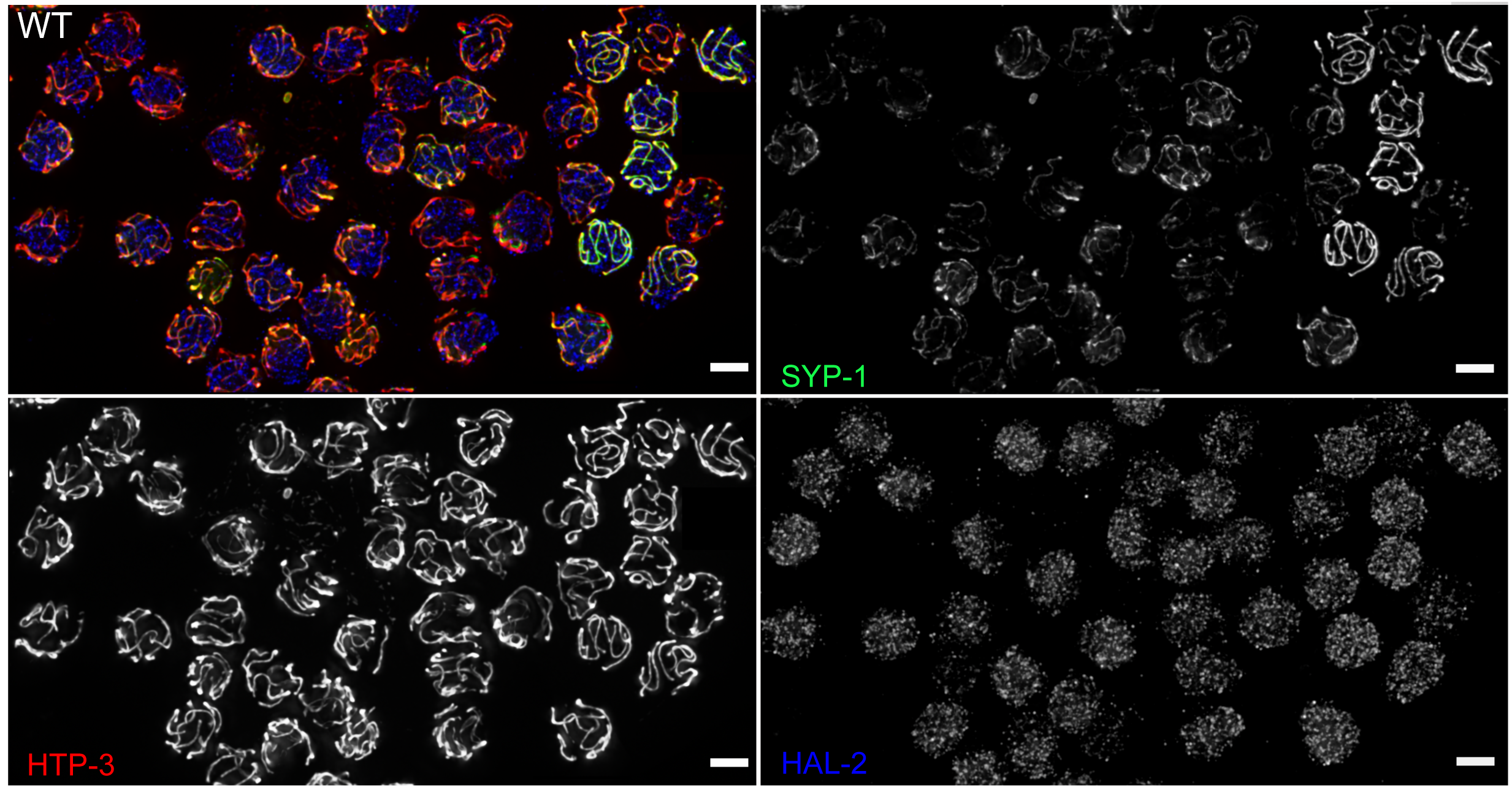

Supplement: S4 Fig — Representative field of nuclei from the early-to-late pachytene transition region of a WT gonad subjected to detergent-based lysis and immunostained to detect localization of HTP-3 (axis), SYP-1 (SC central region) and HAL-2 (a nucleoplasmic protein). As we have observed that nuclei in earlier stages of prophase are in general more susceptible than late prophase nuclei to disruption and spreading by detergent-based lysis procedures, we wanted to rule out the possibility that the higher sensitivity of SYP-1 to removal at earlier stages might be explained solely as a secondary consequence of this higher sensitivity to nuclear disruption. To this end, we carried out simultaneous immunoloclaization of SYP-1 and HAL-2, a nuclear protein that is concentrated in the nucleoplasm but is not associated with chromatin. As HAL-2 is largely, but not completely, released from nuclei when they are partially disrupted by detergent lysis, the amount of residual HAL-2 detected in nuclei can serve as a proxy for the degree of nuclear disruption. In the nuclei depicted here, we did not observe a correlation between the amount of residual HAL-2 and SYP-1 after detergent lysis, indicating that the differential sensitivity of early and late pachytene nuclei to SYP-1 removal can be observed even when the overall degree of nuclear disruption is comparable. Thus, the higher sensitivity of SYP proteins to removal at earlier stages is not solely a secondary consequence of earlier nuclei being more susceptible to mechanical disruption, but rather implies a state change in the SCs themselves. Scale bar, 5μm. (TIF) [file pgen.1006670.s004.tif]

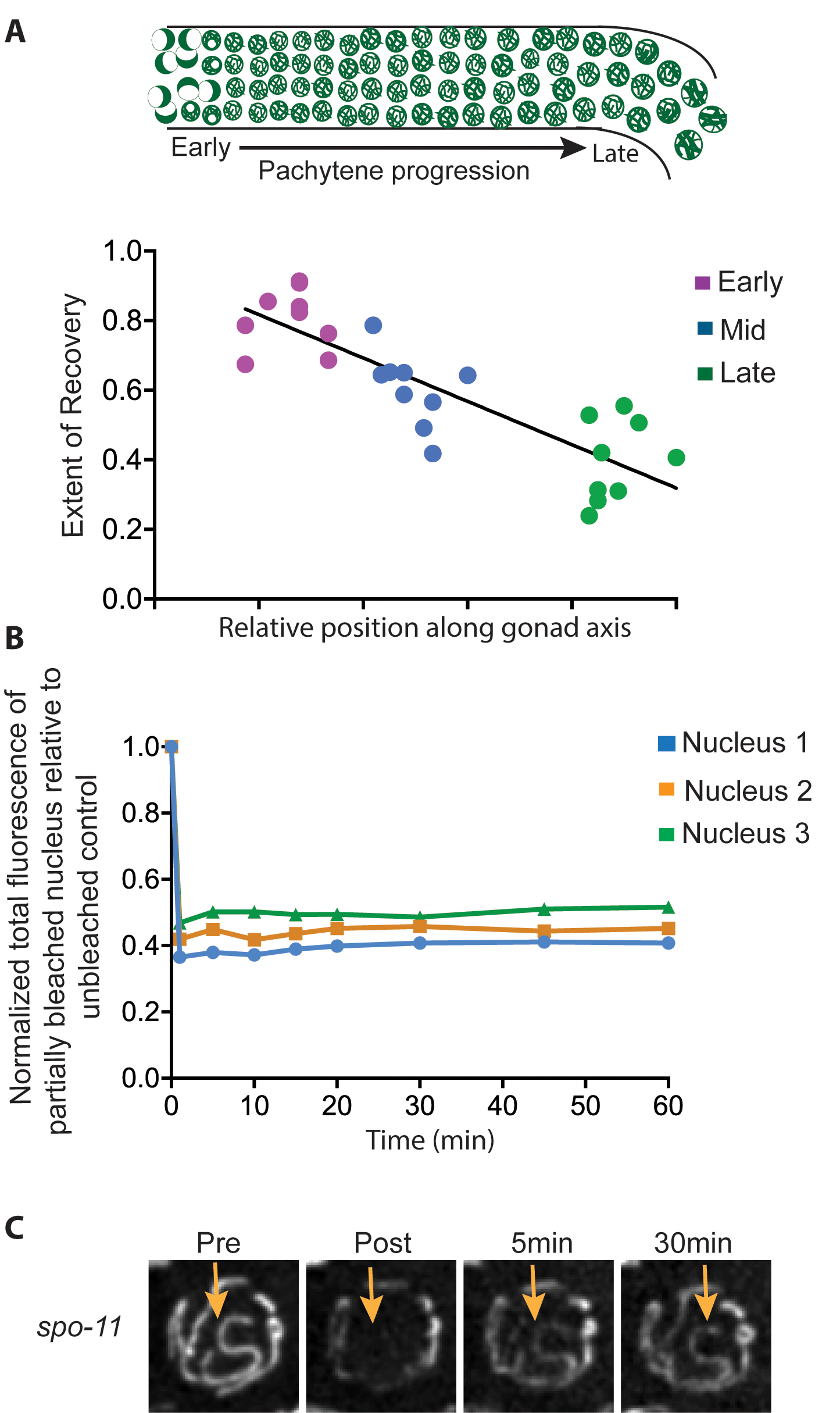

Supplement: S5 Fig — A. Alternative representation of the quantitation of the confocal FRAP experiments presented in Fig 3B. The plateau values for the “extent of fluorescence recovery” curves for each nucleus were plotted against the position of that nucleus along the gonad axis. Relative position along the gonad axis was calculated by dividing the row number of the nucleus by the total number of pachytene rows in that gonad. Slope of best fit line = -0.62 (R2 = 0.71), reflecting a reduction in GFP::SYP-3 dynamics as nuclei progress through the pachytene stage. B. Graph plotting the total fluorescence in representative partially bleached nuclei (relative to an unbleached reference nucleus in the same field) from a FRAP experiment analyzing mid-pachytene nuclei in wild-type worms. Normalized total fluorescence in each partially bleached nucleus remains nearly constant during the recovery time course when compared to an unbleached reference nucleus. C. Similar to Fig 3E: panel of EmGFP::SYP-3 images of a spo-11 mutant nucleus showing FRAP of an SC that had been bleached along its entire length (arrow). (TIF) [file pgen.1006670.s005.tif]

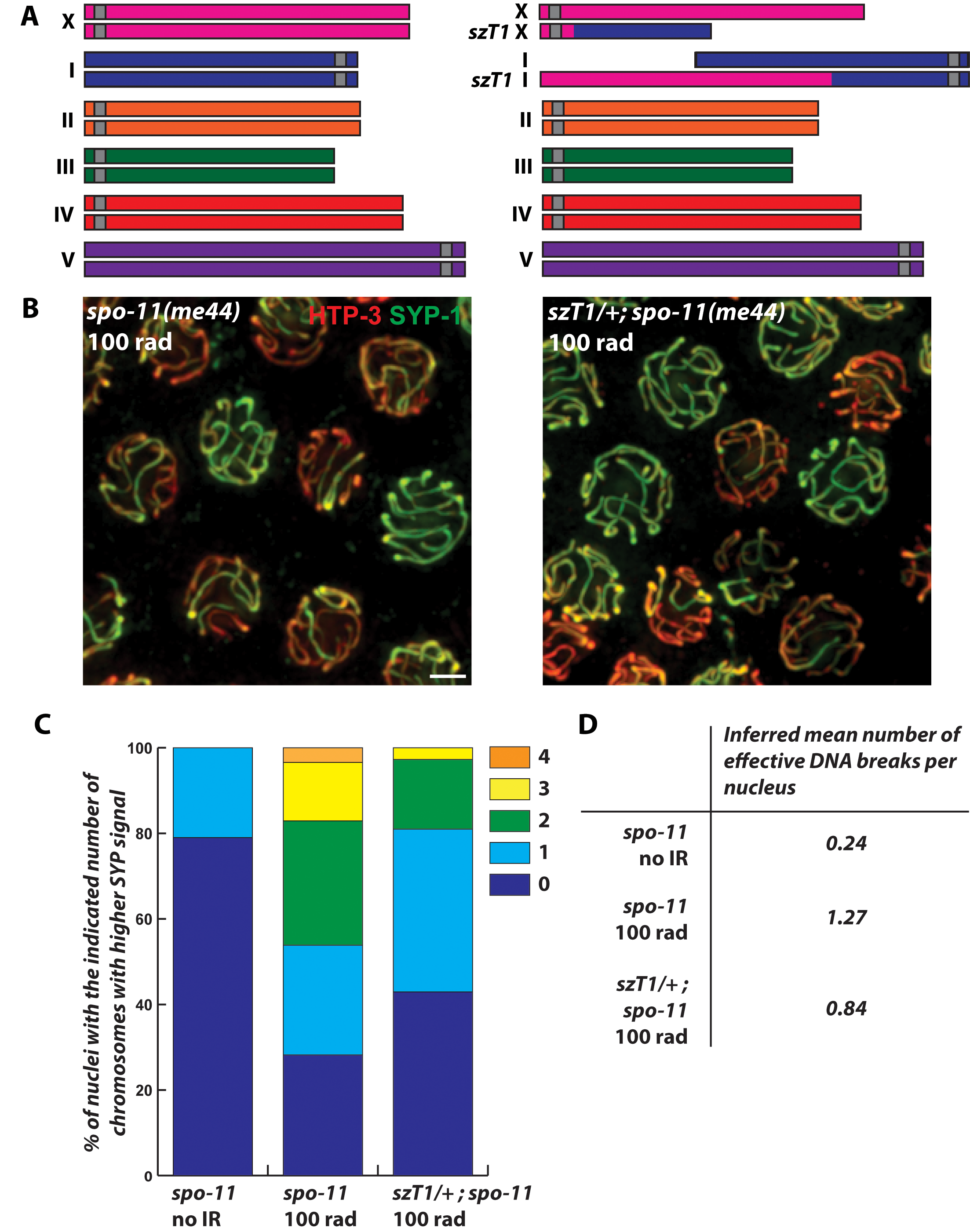

Supplement: S6 Fig — A. Schematic representation of synaptic configurations in spo-11 mutant meiocytes (left) and szT1 heterozygous spo-11 mutant meiocytes (right). The szT1 rearrangement is a reciprocal translocation between chromosomes I and X, and therefore in szT1 heterozygous meiocytes, one third to one quarter of the genome is synapsed non-homologously. B. Representative fields of whole-mount late pachytene nuclei from spo-11 (left) and szT1 heterozygous spo-11 (right), stained for HTP-3 and SYP-1 eight hours after exposure to 100 rad of γ-irradiation, showing an increased incidence of nuclei with uneven SYP-1 distribution. Scale bars represent 2μm. C. Quantitation of the proportions of nuclei in the last quarter of the pachytene region with the indicated number of chromosome pairs exhibiting SYP-1 enrichment in gonads of untreated and irradiated spo-11 worms and irradiated spo-11 worms heterozygous for szT1 (Of note, the samples analyzed for unirradiated spo-11 are the same as in Fig 5, but were reanalyzed to focus exclusively on nuclei in the last quarter of pachytene, where SYP-1 enrichment is more pronounced, enabling quantitation of numbers of SYP-1-enriched chromosome pairs within the subset of nuclei exhibiting uneven SYP-1 distribution.) D. We estimated the mean number of DNA breaks per nucleus for each genotype and irradiation dose analyzed in panel C, based on a) previous work showing that in C. elegans meiocytes lacking SPO-11 induced DSBs, a single DSB can be efficiently converted into a CO recombination intermediate, and b) our hypothesis that enrichment of SYP-1 on a subset of chromosomes in late pachytene nuclei might be triggered by the formation of recombination intermediates. Thus, the mean number of DNA breaks that were effective in eliciting SYP-1 enrichment was inferred from the proportion of nuclei with even SYP-1 distribution (dark blue in panel C, = zero effective breaks), assuming a Poisson distribution. The magnitude of the reduction in inferred mean numb [file pgen.1006670.s006.tif]

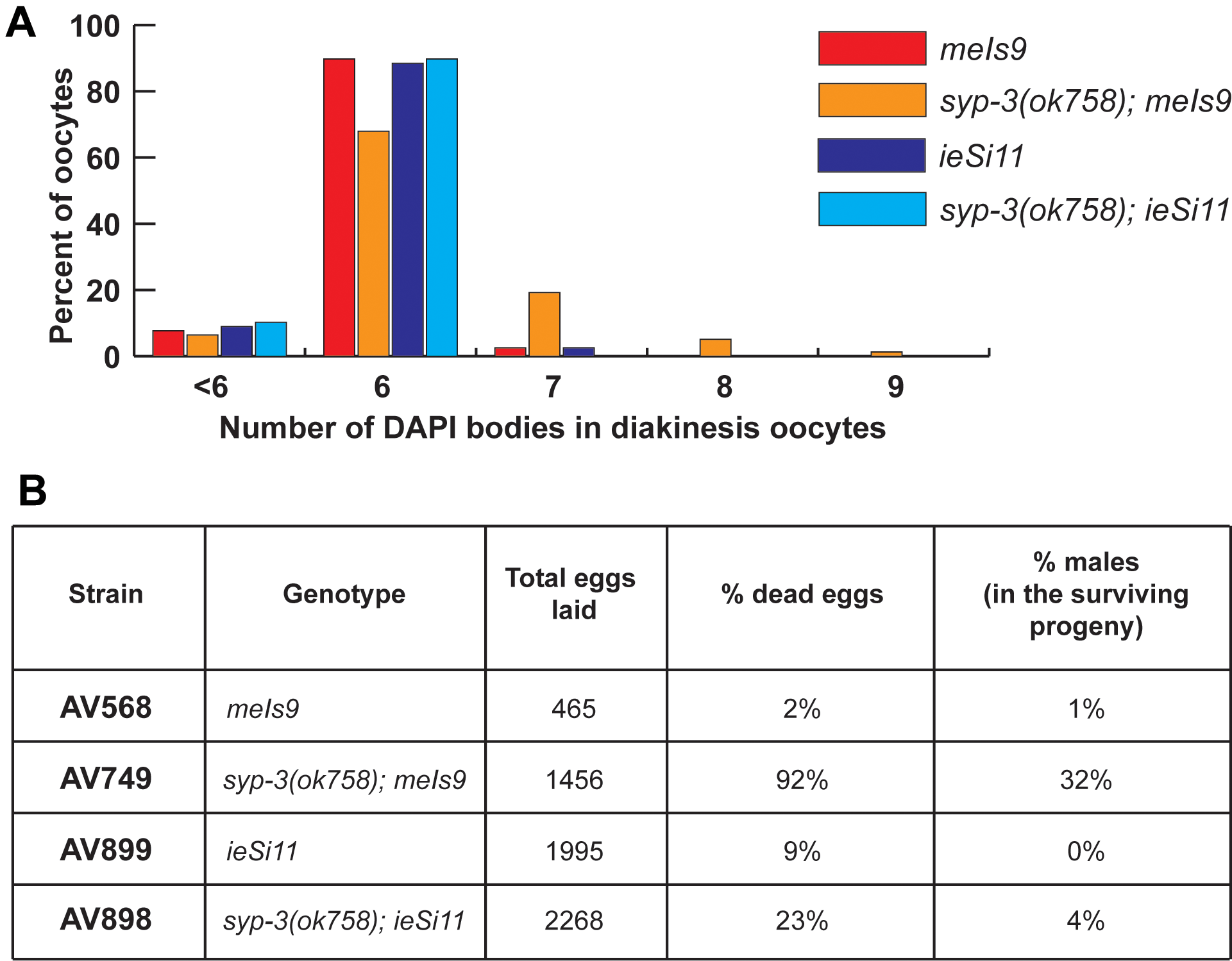

Supplement: S7 Fig — A. Quantitation of DAPI bodies in oocytes at diakinesis, the last stage of meiotic prophase. Wild type oocytes have 6 DAPI bodies, corresponding to six pairs of homologous chromosomes connected by chiasmata; observation of 7–9 DAPI bodies reflects a failure to form chiasmata between a subset of chromosome pairs. 78 nuclei diakinesis nuclei were scored for each genotype. B. Quantitation of percent males among surviving progeny, which reflects mis-segregation of X chromosomes, and inviability of embryos, which in the context of meiotic mutants can reflect mis-segregation of autosomes and/or defective repair of meiotic DSBs. (TIF) [file pgen.1006670.s007.tif]
